# Barriers to Buprenorphine Prescribing for Opioid Use Disorder Among Louisiana Family Physicians: A Pilot Survey Study With Narrative Review

**DOI:** 10.7759/cureus.103848

**Published:** 2026-02-18

**Authors:** Micah Pippin

**Affiliations:** 1 Family Medicine, Louisiana State University Health Sciences Center, Alexandria, USA; 2 Family Medicine, Rapides Regional Medical Center, Alexandria, USA

**Keywords:** buprenorphine, medication-assisted treatment, medication for opioid use disorder (moud), opioid use disorder (oud), substance use disorder

## Abstract

Background

Opioid use disorder is a prevalent and complex condition encountered in primary care. Buprenorphine is widely recognized as an effective pharmacologic treatment, yet it is not routinely incorporated into family medicine practices. This study aimed to assess perceived barriers to buprenorphine prescribing among Louisiana family physicians and to contextualize these barriers through a narrative review focused on practical implementation strategies.

Methods

This pilot, cross-sectional survey study was conducted among practicing family physicians in Louisiana and approved by the Louisiana State University Health Sciences Center-Shreveport Institutional Review Board (IRB). The anonymous, electronic survey was distributed to non-resident members of the Louisiana Academy of Family Physicians (LAFP) in September 2023 using a web-based platform. The survey assessed current buprenorphine prescribing practices and perceived barriers among non-prescribing respondents. Descriptive statistics were used to summarize results.

Results

A total of 65 family physicians completed the survey. Seventeen respondents (26%) reported actively prescribing buprenorphine for opioid use disorder, while 48 (74%) did not. Barrier-related items were completed only by non-prescribers (n = 48). The most frequently reported barriers included concern about attracting disruptive patients (54%), insufficient time to initiate and manage treatment (50%), lack of access to substance use disorder specialists (50%), insufficient education or training in opioid use disorder management (48%), concerns about diversion or misuse (37.5%), and limited availability of mental health services (37.5%). Regulatory concerns, including fear of Drug Enforcement Administration (DEA) intrusion, were reported by 27% of respondents, while fewer expressed concerns regarding safety, effectiveness, reimbursement, or practice partner resistance.

Conclusions

In this pilot survey of Louisiana family physicians, most respondents did not prescribe buprenorphine despite recognizing the ongoing need for opioid use disorder treatment in their communities. Barriers to prescribing were multifactorial and primarily related to time constraints, educational gaps, perceived clinic disruption, and limited support resources rather than doubts about medication safety or efficacy. These findings highlight opportunities for targeted educational, structural, and workflow-focused interventions to expand medication for opioid use disorder (MOUD) delivery in family medicine and improve access to evidence-based opioid use disorder treatment.

## Introduction

Opioid use disorder continues to represent a major public health crisis in the United States, with opioid-related morbidity and mortality remaining unacceptably high despite ongoing prevention and treatment efforts. Louisiana has been disproportionately affected by the opioid epidemic, experiencing sustained increases in overdose-related emergency department visits, hospitalizations, and deaths over the past decade [1]. Expanding access to effective, evidence-based treatment remains a critical component of addressing this ongoing crisis.

Medication for opioid use disorder (MOUD), including buprenorphine, methadone, and extended-release naltrexone, is associated with substantial reductions in all-cause mortality, opioid-related overdose deaths, and illicit opioid use [2-4]. Among these options, buprenorphine is uniquely positioned for use in primary care settings due to its favorable safety profile, partial opioid agonist properties, and feasibility for office-based prescribing [4]. Family physicians in particular are well-positioned to provide longitudinal, comprehensive care for patients with opioid use disorder, especially in rural and underserved communities where access to specialty addiction services may be limited [5].

Despite strong evidence supporting its effectiveness and recent regulatory changes intended to expand access, buprenorphine remains underutilized in primary care [5-7]. Prior studies have identified multiple barriers to buprenorphine prescribing among primary care physicians, including limited training, time constraints, concerns about diversion, regulatory scrutiny, inadequate reimbursement, and lack of access to behavioral health or specialty support [8-16]. However, many of these studies predate recent changes to federal prescribing requirements or focus on regions outside the southeastern United States, limiting their applicability to current practice environments and to Louisiana specifically.

Understanding region-specific and practice-level barriers is essential for designing targeted interventions that meaningfully expand MOUD access. Louisiana presents a unique context due to its high burden of opioid-related harm, mixed urban and rural populations, and ongoing efforts to integrate addiction treatment into primary care [1]. To date, limited data exist describing perceived barriers to buprenorphine prescribing among Louisiana family physicians.

The objective of this pilot study and narrative review was twofold. First, to assess buprenorphine prescribing practices and to characterize perceived barriers to MOUD adoption among practicing Louisiana family physicians. Second, to provide comprehensive guidance on MOUD utilization and implementation, possibly assuaging perceived barrier-related practitioner concerns and promoting an evidence-informed understanding of buprenorphine prescribing. By identifying modifiable educational, structural, and system-level barriers, this study also aims to inform future interventions to support family physicians in expanding access to evidence-based opioid use disorder treatment.

## Materials and methods

This study was a pilot, cross-sectional survey of practicing family physicians in Louisiana and was approved by the Louisiana State University Health Sciences Center-Shreveport Institutional Review Board (IRB). The survey was distributed during September 2023 to physician members of the Louisiana Academy of Family Physicians (LAFP).

Survey distribution was facilitated by the LAFP membership and outreach coordinator using the SurveyMonkey® (Momentive Inc., San Mateo, CA) web-based survey platform. An invitation containing a survey link was emailed to eligible participants, and participation was voluntary. No incentives were offered for survey completion. The survey was anonymous, and no identifying personal or practice-level data were collected.

Eligible participants included all non-resident family physician members of the LAFP. Resident physicians were excluded, as they are currently receiving formal education in opioid use disorder management and MOUD prescribing as part of Accreditation Council for Graduate Medical Education (ACGME) program requirements, which could confound assessment of perceived barriers. There were no additional exclusion criteria.

The survey consisted of 13 items. The first question assessed whether respondents currently prescribe buprenorphine for opioid use disorder as part of their clinical practice. Respondents who answered “no” to this initial question were then directed to complete the remaining 12 questions, which evaluated perceived barriers to initiating or providing buprenorphine treatment.

Survey questions were developed based on a focused literature review conducted through PubMed using the keywords “buprenorphine,” “barriers,” and “family physicians.” Peer-reviewed articles describing commonly reported barriers to buprenorphine prescribing in primary care settings were reviewed and informed the selection of survey items [8-16]. Articles not written in English, not peer-reviewed, or not relevant to barrier identification were excluded. The final survey items reflected recurrent themes identified in the literature, including concerns related to education and training, time constraints, reimbursement, diversion, regulatory scrutiny, community resources, and patient complexity. The complete survey instrument is provided in the Appendices.

Respondents were allowed to select multiple barriers, reflecting the multifactorial nature of prescribing decisions. All survey questions were closed-ended to facilitate descriptive analysis.

Survey responses were collected electronically through SurveyMonkey® and exported for analysis. Because the survey was anonymous and did not collect identifiable information, responses could not be linked to individual participants. There were no missing data, as completion of survey items was required for submission.

Results were summarized using descriptive statistics. Categorical variables were reported as frequencies and percentages. No inferential statistical analyses were performed, as this pilot study was exploratory in nature and designed to characterize perceived barriers and inform future, larger-scale investigations rather than test specific hypotheses. Data analysis was performed using spreadsheet-based software.

All screening and diagnostic tools referenced in this study, including the Opioid Risk Tool (ORT), Current Opioid Misuse Measure (COMM), Clinical Opiate Withdrawal Scale (COWS), and Diagnostic and Statistical Manual of Mental Disorders, Fifth Edition (DSM-5) diagnostic criteria, are publicly available for clinical and research use and do not require licensing fees [17-22].

## Results

A total of 65 family physicians completed the anonymous survey. All survey items were completed in full, and no missing data were observed. No demographic information was collected, and responses were analyzed in aggregate. Respondents were permitted to select multiple perceived barriers where applicable.

**Figure 1:**
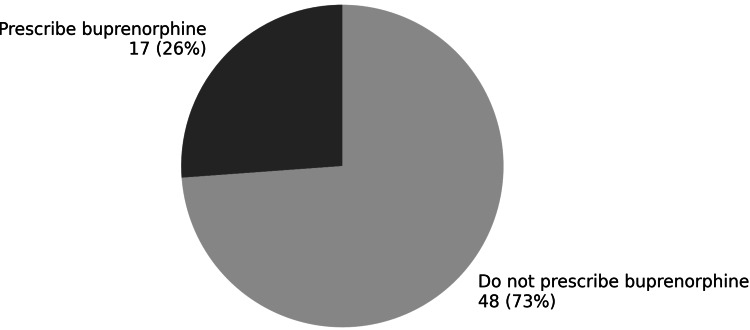
Buprenorphine prescribing status among survey respondents (n = 65) Figure created by the author using Microsoft Word (Microsoft® Corp., Redmond, WA).

Among survey respondents, 17 physicians (26%) reported actively prescribing buprenorphine for opioid use disorder as part of their clinical practice, while 48 physicians (74%) reported that they do not currently prescribe buprenorphine (Figure 1).

Barrier-related survey items were completed only by respondents who reported not prescribing buprenorphine (n = 48). Respondents were permitted to select multiple barriers; therefore, reported percentages do not sum to 100%.

The most commonly reported barriers included concern about attracting drug users or disruptive patients (26 respondents; 54%), lack of available time to initiate and manage buprenorphine treatment (24 respondents; 50%), and lack of available specialists in substance use disorder to assist with complex patients (24 respondents; 50%). Additional frequently cited barriers included insufficient education or background in opioid use disorder management (23 respondents; 48%), concerns regarding diversion or misuse of buprenorphine (18 respondents; 37.5%), and limited availability of mental health services in the community (18 respondents; 37.5%).

**Figure 2:**
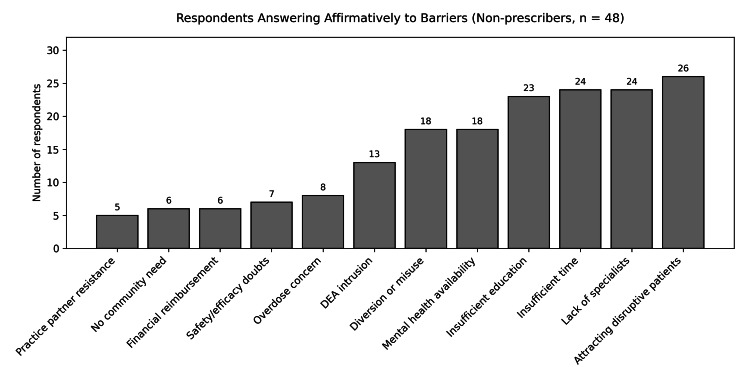
Respondents reporting barriers to buprenorphine prescribing among non-prescribing Louisiana family physicians Figure derived from survey data collected from Louisiana family physicians (September 2023); survey items informed by previously published primary care buprenorphine barrier analyses [8-16]. Figure created by the author using Microsoft Word (Microsoft® Corp., Redmond, WA).

Less commonly reported barriers included concerns about Drug Enforcement Agency (DEA) intrusion (13 respondents; 27%), patient overdose on prescribed buprenorphine (eight respondents; 17%), doubts regarding safety or effectiveness of buprenorphine therapy (seven respondents; 14.5%), concerns about financial reimbursement (six respondents; 12.5%), perceived lack of community need for opioid use disorder treatment (six respondents; 12.5%), and anticipated practice partner resistance (five respondents; 10%) (Table 1 and Figure 2).

**Table 1:** Perceived barriers to buprenorphine prescribing among non-prescribing Louisiana family physicians Respondents could select multiple barriers; percentages use non-prescribers (n = 48) as the denominator. Data derived from an original survey of Louisiana Academy of Family Physicians members (September 2023), informed by previously published barriers literature [8-16].

Perceived barrier	Respondents, n (%)
Concern about attracting drug users or disruptive patients	26 (54.2%)
Insufficient time to initiate and manage buprenorphine treatment	24 (50.0%)
Lack of available substance use disorder specialists	24 (50.0%)
Insufficient education or training in opioid use disorder management	23 (47.9%)
Concerns regarding diversion or misuse of buprenorphine	18 (37.5%)
Limited availability of mental health services	18 (37.5%)
Concern about Drug Enforcement Administration (DEA) intrusion	13 (27.1%)
Concern about patient overdose on prescribed buprenorphine	8 (16.7%)
Doubts regarding safety or effectiveness of buprenorphine therapy	7 (14.6%)
Financial reimbursement concerns	6 (12.5%)
Perceived lack of community need for opioid use disorder treatment	6 (12.5%)
Anticipated practice partner resistance	5 (10.4%)

## Discussion

Epidemiologic context and rationale

**Figure 3:**
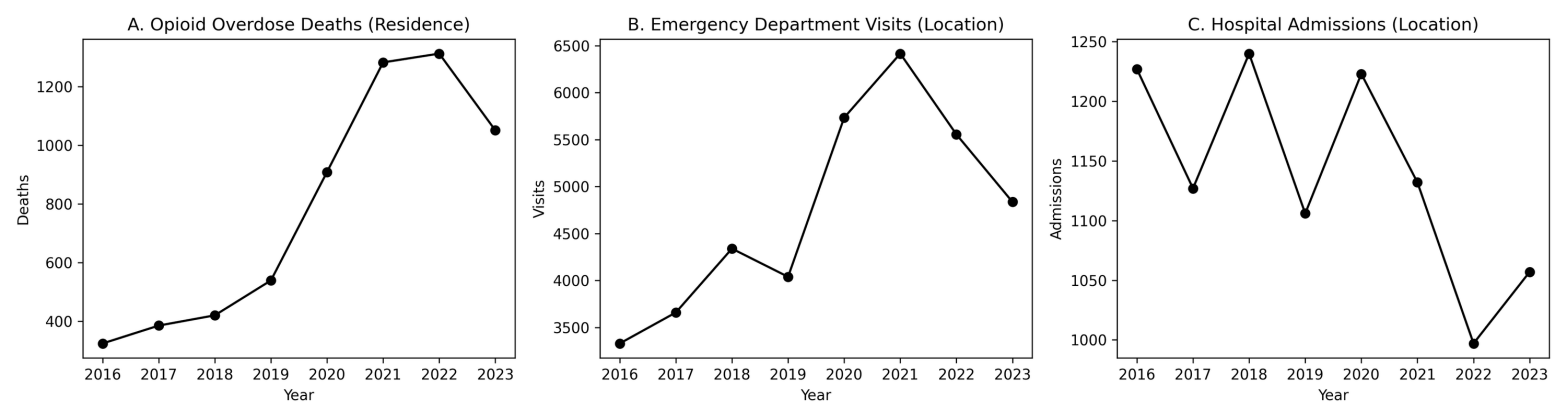
Trends in opioid-related outcomes and healthcare utilization in Louisiana (2016-2023). (A) Annual opioid deaths by patient residence. (B) Annual opioid-related emergency department visits by location of treatment. (C) Annual opioid-related hospital admissions by location of treatment. Figure created by the author using Microsoft Word (Microsoft® Corp., Redmond, WA) and Adobe Acrobat (Adobe Inc., San Jose, CA) and adapted from the Louisiana Department of Health Opioid Data Surveillance System [23].

The opioid epidemic in the United States continues to be a public health crisis. While statistics vary, the Centers for Disease Control and Prevention (CDC) has reported yearly increases in opioid overdose mortalities peaking in 2022 with an estimated 84,181 deaths [1]. In 2023, the first decrease in national opioid-related deaths since 2018 was observed, with an estimated 81,083 deaths [1]. The State of Louisiana is among the most affected by continuing opioid-related morbidity and mortality [23]. The Louisiana Department of Health, through its opioid data and surveillance system, has recorded trends congruent with national statistics, with opioid-related deaths peaking in 2022 and decreasing for the first time in 2023 (Figure 3A) [23]. A similar trajectory can be seen in opioid overdose-related emergency department visits in Louisiana, with encounters increasing significantly over the last decade and dipping in 2023 (Figure 3B) [23]. Louisiana hospital admissions for opioid overdoses have generally remained consistent in the same reported time period (Figure 3C) [23].

**Figure 4:**
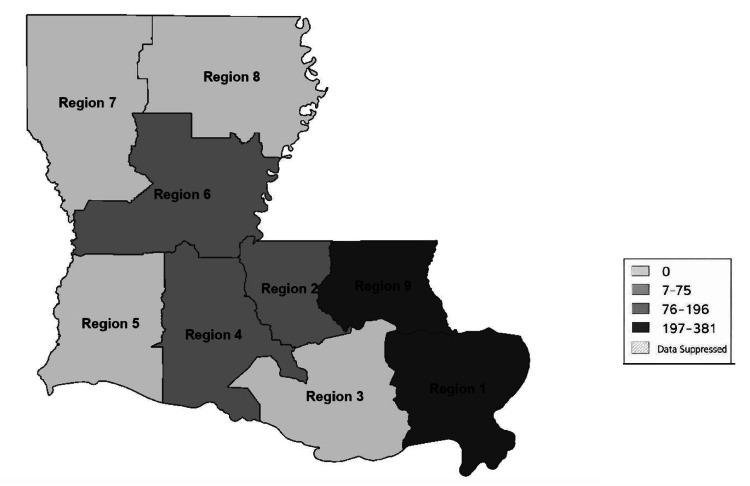
Opioid overdose deaths by the Louisiana Department of Health region (2023) Figure created by the author using PowerPoint (Microsoft® Corp., Redmond, WA) and Adobe Acrobat (Adobe Inc., San Jose, CA) and adapted from the Louisiana Department of Health Opioid Data Surveillance System [23].

Only a small number of survey respondents, 12.5%, reported a lack of need for opioid use disorder management in their Louisiana community; however, the problem persists throughout the state and country, and no location or demographic is immune to the effects of the opioid epidemic. The geographical representation of 2023 opioid overdose deaths in the state of Louisiana by region demonstrates a heterogeneous distribution of communities affected by opioid related mortality, including rural and urban settings (Figure 4) [23].

**Figure 5:**
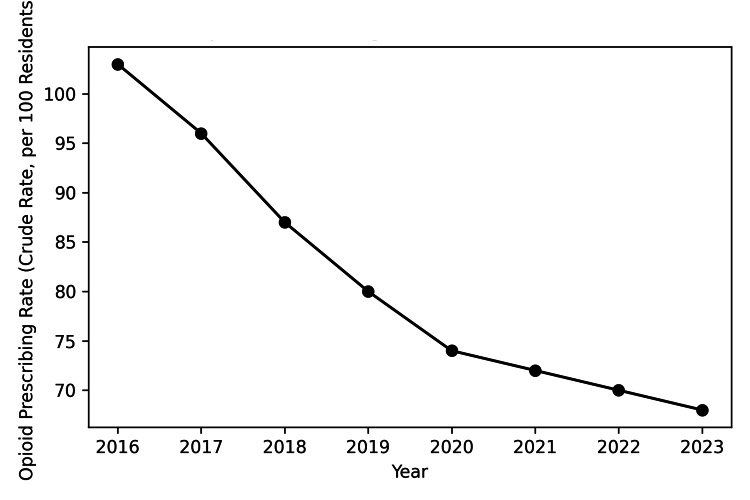
Annual opioid prescribing rates in Louisiana (2016-2023) Figure created by the author using Microsoft Word (Microsoft® Corp., Redmond, WA) and adapted from the Louisiana Department of Health Opioid Data Surveillance System [23].

Practitioners have sought to address the problem by limiting opioid analgesic prescriptions dispensed. In Louisiana, the number of opioids prescribed has consistently decreased from 2016 to 2023 (Figure 5) [23].

However, today’s opioid deaths are more commonly associated with unregulated, non-prescription synthetic opioids such as fentanyl and its analogues [1-4]. A non-prescribing strategy has not resulted in improvement in patient and community outcomes and reflects the need for proactive interventions [23]. To properly combat this epidemic, family physicians must adopt evidence-based strategies, including pharmacotherapeutic interventions.

Policy environment and regulatory considerations

MOUD, formerly known as medication-assisted treatment (MAT), remains a proven therapy for opioid use disorder with demonstrated decreases in opioid deaths, improved retention in management programs, and sustained abstinence from opioids [2-4]. The nomenclature change from MAT to MOUD reflects a destigmatized and evolving perception of opioid use disorder management, with pharmacotherapy taking a central rather than adjunctive role [4]. Increased utilization of buprenorphine and eliminating barriers to its prescribing are targets for numerous entities attempting to curb the devastating effects of the opioid epidemic. A significant step towards improving access to buprenorphine was the Medication Access and Training Expansion (MATE) Act, passed by Congress in December 2023, which allowed all providers with a DEA license to prescribe buprenorphine as a treatment for opioid use disorder [24-26]. Prior to this, office-based buprenorphine prescribing outside of official opioid treatment facilities was limited to practitioners with an X waiver obtained through special training programs and with restrictions on the total number of patients who could be managed [24-26].

During the X waiver era, a 2020 Medicare claims study observed that family medicine physicians accounted for the highest volume of buprenorphine prescribers from 2013 to 2016; however, only 2.4% of family physicians were prescribing buprenorphine [27]. These numbers indicate an opportunity for the specialty of family medicine to improve access to MOUD and better address the opioid epidemic. While the MATE Act removed a major roadblock to the expansion of buprenorphine utilization, additional barriers remain requiring further investigation. According to the CDC, national buprenorphine dispensing rates have not significantly improved from 2019 to now, with 4.7 buprenorphine prescriptions being dispensed per 100 persons in 2023 [28]. In Louisiana, the CDC reports that buprenorphine prescribing has continued to decrease from 5.9 prescriptions per 100 persons in 2019 to 5.4 prescriptions per 100 persons in 2023 [28].

A downstream effect of the MATE Act and the elimination of the X waiver is decreased oversight and scrutiny from regulatory institutions [24-26]. One common concern among family physicians, including 27% of those surveyed in this study, is increased intrusion from the DEA or other regulatory bodies if opioid use disorder treatment is undertaken in primary care settings. Now, prescribing buprenorphine for opioid use disorder is no different than prescribing any other schedule III drug, and the patient caps and training requirements of the X waiver period have been removed [24-26]. Regulatory efforts from organizations like the DEA are more focused on the prevention of unsafe or fraudulent prescribing practices and are supportive of evidence-based interventions to address the opioid epidemic, such as buprenorphine prescribing [24-26]. In fact, engagement in structured MOUD programs may actually reduce regulatory risk by stabilizing prescribing patterns and improving documentation consistency [24-26].

Screening, diagnosis, and identification of opioid use disorder

Identifying patients who could benefit from MOUD is an initial step in the pathway for implementing effective opioid use disorder management and curbing the opioid epidemic. In 2020, the United States Preventive Services Task Force (USPSTF) proposed that adults 18 years or older should be screened for unhealthy drug use with a grade B level recommendation [29]. The American Academy of Family Physicians (AAFP) has concluded that there is insufficient evidence (I recommendation) to screen all asymptomatic adults for unhealthy drug use; however, they offer a grade C recommendation for selectively screening adults 18 years and older specifically for opioid use disorder [30]. Both organizations agree that screening should only take place if there are available services for accurate diagnosis, effective treatment, and appropriate care [29-30]. With the X waiver and now the MATE Act, family physicians can screen for, diagnose, and effectively manage patients with opioid use disorder [24-26].

There are several validated screening tools available for opioid use disorder risk assessment, including the COMM and the ORT [17-18,20]. The DSM-5 offers a set of diagnostic criteria written by the American Psychiatric Association (APA) for confirming and measuring the severity of opioid use disorder (Table 2) [22].

**Table 2:** Diagnostic and Statistical Manual of Mental Disorders, Fifth Edition (DSM-5) diagnostic criteria for opioid use disorder Table created by the author based on available DSM-5 criteria [22].

Criteria
Opioids are often taken in larger amounts or over a longer period of time than intended.
There is persistent desire or unsuccessful efforts to cut down or control opioid use.
A great deal of time is spent in activities necessary to obtain the opioid, use the opioid, or recover from its effects.
Craving, or a strong desire to use opioids.
Recurrent opioid use resulting in failure to fulfill major role obligations at work, school, or home.
Continued opioid use despite having persistent or recurrent social or interpersonal problems caused or exacerbated by the effects of opioids.
Important social, occupational, or recreational activities are given up or reduced because of opioid use.
Recurrent opioid use in situations in which it is physically hazardous.
Continued use despite knowledge of having a persistent or recurrent physical or psychological problem that is likely to have been caused or exacerbated by opioids.
Tolerance, as defined by either of the following: (a) a need for markedly increased amounts of opioids to achieve intoxication or desired effect; (b) markedly diminished effect with continued use of the same amount of an opioid.
Withdrawal, as manifested by either of the following: (a) the characteristic opioid withdrawal syndrome; (b) the same (or closely related) substances are taken to relieve or avoid withdrawal symptoms.

Patient selection and clinical complexity

Once a diagnosis is established, appropriate patient selection is essential to successfully implementing MOUD in the primary care setting. Individuals with well-controlled somatic and psychiatric conditions, a stable and substance-free home environment, and without the use of other sedatives such as benzodiazepines are ideal candidates for management in the family medicine clinic [2-4]. Still, the use of prescribed benzodiazepines or stimulants should not definitively preclude patients with opioid use disorder from receiving appropriate treatment with buprenorphine [31]. Evidence is mixed on the overall effect on outcomes from buprenorphine and benzodiazepine/stimulant coadministration. Some studies report a small increased overdose risk with concurrent use, and others describe improved treatment retention in opioid use disorder programs when benzodiazepines or stimulants are continued with buprenorphine [31]. The decision to initiate MOUD in patients currently prescribed benzodiazepines or stimulants should be individualized, recognizing that withholding buprenorphine treatment is not recommended, while also acknowledging the increased clinical complexity that co-prescribing entails [31]. Conversely, MOUD with methadone prescribed alongside benzodiazepines or stimulants is associated with increased overdose risk and mortality and should be approached with caution [31].

Buprenorphine pharmacology, safety, and overdose risk

Buprenorphine’s pharmacology as a partial opioid agonist is responsible for its effectiveness in managing opioid use disorder [2-4]. As a partial agonist, its intrinsic activity at the mu receptor is low, making it less potent and creating a ceiling effect, which significantly decreases the risk of respiratory depression compared to full opioid agonists [2-4]. Buprenorphine also has a much higher affinity for mu receptors than full opioid agonists [2-4]. This powerful attraction allows buprenorphine to displace other opiates and anchor to the receptors, preventing its own displacement in the continued presence of full agonists [2-4]. Essentially, buprenorphine can reverse or prevent the action of drugs like morphine and heroin while maintaining the patient’s respiratory effort and avoiding withdrawal symptoms. Understanding this pharmacology is essential to addressing barriers associated with efficacy and safety concerns. Of those family physicians queried in our study, 14.5% reported doubts about the effectiveness and/or safety of buprenorphine therapy for opioid use disorder. Seventeen percent of respondents answered affirmatively to being concerned about a patient overdosing on a buprenorphine prescription that they provided. An appreciation of the underlying pharmacodynamics of buprenorphine and its receptor-level effects can help assuage fears of inefficacy or danger. The combination of its partial agonist ceiling effect on respiratory depression and its competitive blockade of full opioids makes for an ideal, practical, and safe therapeutic choice for treating opioid use disorder [2-4]. Overdoses associated with buprenorphine are rare and are most associated with concurrent sedative use, including supratherapeutic doses of benzodiazepines and other central nervous system depressants such as alcohol [2-4]. Opioid naïve patients may also be more prone to buprenorphine overdose; however, this is still uncommon [2-4].

Buprenorphine/naloxone formulation, pregnancy, and special populations

In 2002, a formulation of buprenorphine combined with naloxone (Suboxone) was released in the United States as a combination product that could decrease the risk of misuse and abuse of buprenorphine [2-4]. Naloxone is an opioid receptor antagonist that can be utilized to reverse the effects of opioid agonists [2-4,32]. In its sublingual and oral forms, such as in Suboxone, naloxone has relatively low bioavailability compared to buprenorphine and would have minimal, if any, effects on opioid mu receptors [32]. However, if these preparations were crushed or dissolved and administered parenterally through nasal inhalation or intravenous injection, the naloxone would override buprenorphine’s effect and induce opioid withdrawal symptoms [32]. These pharmacokinetic properties are the basis for the addition of naloxone to buprenorphine to act as a deterrent for misuse and diversion [2-4,32]. Recent investigations have questioned the utility of adding naloxone to buprenorphine. While naloxone does have a high affinity for mu opioid receptors when bioavailable, buprenorphine’s affinity is even higher [31]. This, combined with buprenorphine’s slow receptor dissociation kinetics and naloxone’s rapid elimination, suggests that the effects of parenteral naloxone in the presence of buprenorphine would be minimal and short-lived [32]. Recent investigations have demonstrated no difference in mortality between groups taking buprenorphine monotherapy and combined buprenorphine/naloxone; however, epidemiologic studies do show a decrease in parenteral misuse after the introduction of the combined formulation [32]. Despite the controversy, the administration of compounded buprenorphine/naloxone is currently recommended over the use of buprenorphine monotherapy for opioid use disorder, except in certain cases, such as an allergy to naloxone [2-4,32].

In pregnancy, guidance for opioid use disorder treatment has traditionally included avoidance of the buprenorphine/naloxone combined formulation in favor of buprenorphine alone, as the safety profile of naloxone in pregnancy had not been fully established [2-4,33-35]. However, recent investigations have demonstrated similar, and sometimes improved, maternal and neonatal outcomes with the use of buprenorphine/naloxone compared to buprenorphine monotherapy [33-35]. Evidence has also supported buprenorphine/naloxone as a safer alternative to methadone for MOUD in pregnancy, with Ordean et al. reporting reduced rates of neonatal abstinence syndrome requiring pharmacotherapy when buprenorphine/naloxone was implemented over methadone [35]. These recent investigations have affirmed that combination buprenorphine/naloxone is a safe and reasonable alternative to buprenorphine monotherapy in pregnancy [33-35].

While MOUD availability for adults is expanding, adolescents with opioid use disorder typically do not access pharmacologic therapies and, if treatment is engaged, undertake abstinence-based programs and psychosocial support interventions that are more susceptible to relapse and dropout [36]. Even when MOUD is provided to adolescents, they often receive highly monitored and abbreviated detoxification courses that are subject to increased treatment failure [36]. Unlike methadone, buprenorphine is approved for the management of opioid use disorder in adolescents as young as 16 [36]. Randomized controlled trials investigating the efficacy of buprenorphine in younger patients have found that, as in adults, longer treatment durations and more flexible home-administration schedules are associated with improved treatment retention [36]. Continued research into optimal management models for adolescents with opioid use disorder is needed; however, the available evidence suggests significant parallels between effective adult and adolescent buprenorphine treatment principles [36].

Formulations, induction, and workflow integration

Buprenorphine and buprenorphine/naloxone are typically administered as sublingual films or tablets, taken daily, twice daily, or three times daily [2-4]. An extended-release buprenorphine six-month subdermal implant was approved for use in 2016; however, this preparation was discontinued due to cost [2-4]. Subcutaneous sustained-release injectable buprenorphine is available and is administered weekly to monthly, depending on the formulation [2-4].

Before starting MOUD with buprenorphine, a baseline urine drug screen (UDS) should be collected [2-4].

To facilitate informed consent and establish goals, objectives, standards, and expectations, a patient-physician contract should be signed prior to initiation of MOUD [2-4]. These agreements are unique to individual facilities; however, the American Society of Addiction Medicine (ASAM) does provide a suggested outline [2-4]. Topics such as safe storage, accountability, behavior, counseling and support, communication, adherence, and dosing are common components of opioid use disorder treatment contracts and set a precedent for the patient-physician relationship and future interactions [2-4].

Initiating buprenorphine therapy for opioid use disorder is known as induction and is tasked with minimizing withdrawal symptoms, achieving an effective maintenance dose, and avoiding precipitated withdrawal [21,37]. When full opioid agonists are still present in circulation and bound to mu receptors, administering buprenorphine can displace the attached full agonist and instigate significant withdrawal symptoms [21,37]. For this reason, patients are instructed to prepare for induction by abstaining from opiates for eight to 12 hours, placing them in mild to moderate withdrawal, and avoiding more significant precipitated withdrawals [2-4,21,37]. Induction practices are varied and have evolved over the last decade. Beginning in the infancy of buprenorphine prescribing, the standard of care was office-based induction. Following a preparatory visit where an individual’s suitability for MOUD was established and a buprenorphine contract was signed, patients returned for induction during a second clinic visit in moderate withdrawal [36-37]. The patient had to retrieve a preliminary prescription from the pharmacy, or the physician had to maintain a supply of buprenorphine in the office [18,37]. Monitored initiation and titration of buprenorphine commenced during this visit and often lasted two to four hours [21,37]. Validated opiate withdrawal scales, such as the COWS, were utilized, and doses of buprenorphine were increased by 2 mg until resolution of withdrawal symptoms [18,21,37]. A subsequent follow-up visit in one to two days is often followed by further dosing adjustments and achievement of maintenance dosing [21,37]. Later, an unobserved patient-centered home-based induction was proposed, which would alleviate several limitations to buprenorphine induction in the office setting, such as patient discomfort with public withdrawal and time demands of busy office healthcare workers, as well as empower patients with a more personal management of their opioid use disorder [21,37]. Several investigations into home-based buprenorphine induction concluded that this method was equally effective to office-based management and was preferred by patients [21,37]. Today, both office-based and home inductions are accepted as appropriate and safe [2-4,21,37].

Relevantly, half of the survey respondents affirmatively answered that time constraints were a perceived barrier to initiating buprenorphine therapy in their practice. The home-based self-administration model essentially eliminates the time-consuming elements of MOUD and is consistent with best practices for patients with opioid use disorder [21,37]. Subsequent chronic buprenorphine management encounters are similar in duration to typical outpatient visits and are not a burden for busy physician schedules [21,37]. Also, unsupervised home-based induction forgoes the need for maintaining an office supply of buprenorphine, further decreasing the likelihood of intrusive regulatory oversight [21,37].

Dosing and maintenance strategies

Buprenorphine may be dosed alone or in combination with naloxone. Sublingual strips and tabs are the most common preparations and are available in 2, 4, 8, and 12 mg buprenorphine doses, with the naloxone concentrations increasing for each higher buprenorphine amount [2-4]. Combination dosage forms are labeled as buprenorphine/naloxone 2 mg/0.5 mg, 4 mg/1 mg, 8 mg/2 mg, and 12 mg/3 mg [2-4]. Patient doses have traditionally been individualized and titrated to an effective dose for the individual patient [2-4,21,37]. One Cochrane review found that all dosing regimens are effective at retaining patients in therapy, with increasing success with higher doses [38]. This study revealed a number needed to treat for buprenorphine to maintain patients in therapy of four for low-dose regimens (2-6 mg), three for medium-dose regimens (7-16 mg), and two for high-dose regimens (16 mg or more) [38]. However, the same review found that only high-dose buprenorphine therapy prevented illicit drug use during treatment [38]. Based on United States Food and Drug Administration (FDA) drug information, the maximum daily dose of transmucosal buprenorphine has been perceived to be 24 mg for some time, as labeling has stated dosages above 24 mg are no more effective than lower doses, and titration goals should seek to achieve no more than 16 to 24 mg daily [39-41]. Researchers and advocates for buprenorphine as treatment for opioid use disorder have asserted that daily doses above 24 mg may be needed to adequately address symptoms in patients who have taken potent synthetic opiates like fentanyl [39-41]. There is emerging, yet limited, evidence on the effectiveness of doses above 24 mg daily [39-41]. One cross-sectional retrospective analysis found that stable buprenorphine doses above 24 mg daily may decrease the risk of subsequent emergency department and inpatient utilization [39]. The FDA has since agreed with buprenorphine proponents and has amended labeling to avoid misinterpretation that there is a maximum transmucosal buprenorphine daily dose of 24 mg [42].

Patient-oriented outcomes and duration of therapy

Patient-oriented outcomes associated with buprenorphine for opioid use disorder include reduced all-cause and overdose mortality, improved treatment retention, reduced illicit opioid use, reduced emergency department visits and hospitalizations, and improved quality of life and functional outcomes [2-4]. The extent to which buprenorphine decreases patient mortality is not absolutely known. One retrospective cohort study observed a 62% decrease in opioid-involved overdose deaths for patients treated with buprenorphine after a non-fatal opioid-involved overdose [43]. The optimal duration of therapy is also controversial. Increased all-cause mortality, suicide, and overdose risk are seen after discontinuing MOUD with buprenorphine [44-45]. A multi-site cohort study found that in patients who discontinue buprenorphine therapy, no specific treatment duration was associated with a reduction in post-treatment mortality [44]. The investigation did conclude that shorter durations of buprenorphine therapy were associated with an increased opioid overdose risk after medication cessation and that that risk does not begin to diminish until at least 12 months of treatment [44]. These and similar findings have led to the assertion that there is no optimal time when buprenorphine can be safely stopped, and many patients should be retained in treatment indefinitely [44-45]. Informally, the recommendation of at least 12 months duration of treatment is proposed [30]. If an informed patient wishes to discontinue buprenorphine, a structured slow-taper schedule should be undertaken with assistance for withdrawal and craving symptoms [44-45]. Overall, few medications prescribed in primary care clinics offer the same degree of near-term mortality reduction and improved patient-oriented outcomes as buprenorphine, including the most commonly prescribed therapies aimed at preventing long-term complications of chronic diseases such as diabetes, hypertension, and dyslipidemia [2-4]. This framing may help answer many family physicians’ questions about the efficacy of buprenorphine and eliminate barriers associated with that uncertainty.

Comparison with methadone

Conflicting data are available comparing the efficacy of buprenorphine with its full opioid agonist counterpart, methadone [46-47]. Both are accepted as first-line pharmacologic interventions for opioid use disorder and are classified as MOUD [2-4,46-47]. Theoretically, the pharmacology of buprenorphine as a partial opioid agonist with a ceiling effect would make it a safer therapeutic option compared to the full agonist methadone. One 2018 primary care cohort study in the United Kingdom demonstrated a decreased all-cause and drug-related poisoning mortality during treatment with buprenorphine compared to methadone, especially in the first four weeks of treatment [47]. However, treatment retention, essential for continued benefit, has been found to be lower in buprenorphine than methadone therapy in this study and others [46-47]. No definitive declaration can be made as to the superiority of one MOUD modality versus the other; however, it is generally accepted that buprenorphine may be more appropriate for uncomplicated mild to moderate opioid dependence and methadone for more severe disease [46-47]. For primary care physicians, prescribing buprenorphine is the more practical and accessible option of the two, as methadone for the management of opioid use disorder must be distributed by a certified methadone clinic through specialized practitioners [46-47].

Laboratory monitoring and infectious disease integration

Preliminary laboratory analysis for patients initiating buprenorphine may include a hepatic function panel or complete metabolic panel (CMP). Buprenorphine therapy has been associated with transaminitis, although it is mostly mild and transient [48-49]. One randomized controlled trial in 2013 found no significant evidence for liver damage during the first six months of treatment with buprenorphine [49]. Instances of severely elevated liver enzymes while receiving buprenorphine are rare and have been mostly associated with patients who are hepatitis C or B positive, are concurrently using illicit drugs, or who inappropriately use buprenorphine by manipulation and intravenous injection [48-49]. Even in reported cases of significant liver injury, the damage appears to be reversible [48-49]. Due to the increased risk of liver injury in patients with previous infectious hepatic diseases, such as hepatitis C and B, it may be prudent to test for these conditions prior to buprenorphine induction; however, it is not required [48-49]. There is no guidance on periodic monitoring of liver function during MOUD; still, practitioners should remain aware of potential adverse reactions such as hepatotoxicity.

The ASAM recommends screening individuals initiating an opioid use disorder treatment program for infectious diseases such as hepatitis C, hepatitis B, and human immunodeficiency virus (HIV) [50-51]. Injection drug use is the leading cause of new hepatitis C infections and is also associated with the contraction of HIV [50-51]. The incidence of hepatitis C has grown with the opioid epidemic and is the leading cause of infection-related mortality in the United States [50-51]. While it is known that opioid use disorder results in a higher risk of acquiring hepatitis C and HIV, the reciprocal is also true, and these infectious diseases can precipitate the development of drug use disorders [50-51]. Testing for these conditions is prudent, as research has demonstrated improved outcomes related to hepatitis C and HIV in patients who are concurrently infected and have opioid use disorder and receive MOUD with buprenorphine [50-51]. The use of MOUD in patients with HIV has been associated with increased antiretroviral therapy (ART) initiation and improved viral suppression [50-51]. Buprenorphine use and retention in opioid use disorder treatment programs have been linked to improved cure rates for hepatitis C [50-51]. Transmission rates of hepatitis C and HIV are also decreased by the pharmacologic treatment of opioid use disorder [50-51]. Management of both these interdependent conditions has surreptitiously become more accessible to primary care physicians in recent years with the advent of buprenorphine and newer simplified direct-acting antiviral regimens targeting hepatitis C [50-51]. Family doctors are ideally placed to provide integrated services combining screening and management of both opioid use disorder and infectious diseases, resulting in increased patient access and compounded improvement in outcomes.

Follow-up, diversion, relapse management, and harm reduction

After a buprenorphine maintenance dose is achieved, a follow-up schedule must be established. Initial weekly or biweekly visits can be lengthened to monthly encounters, even up to three-month extended intervals in patients who have demonstrated stability and compliance [2-4]. Periodic urine drug screening and review of the prescription drug monitoring program (PDMP) are essential components of comprehensive follow-up and treatment monitoring [2-4,52-53]. More frequent UDSs are performed early following induction, then may transition to random drug screening intervals based on individual patient risk and compliance [52-53]. Qualitative immunoassay UDSs are often performed; however, interpretation of results requires awareness of the test’s limitations, susceptibility to false positives and negatives, and the clinical context of the individual patient [52-53]. Quantitative chromatographic urine drug assays are more reliable and can be implemented to confirm results and assess for sample adulteration through buprenorphine metabolites [52-53]. Directly supervised random pill or film counts may be appropriate in cases where diversion is suspected [52-53].

Concerns regarding diversion remain a deterrent to MOUD engagement, and 37.5% of our surveyed Louisiana family physicians listed diversion consequences as a real barrier to personal prescribing of buprenorphine. Diversion is not an intended or acceptable outcome of treatment, and appropriate mitigation strategies must be implemented to counter misuse. However, the public health implications of diverted buprenorphine differ substantially from those of diverted full opioid agonists. Non-prescribed buprenorphine use occurs most commonly in the context of self-treatment of withdrawal symptoms and attempts at illicit drug use cessation rather than for euphoric effect [54-56]. Also, buprenorphine’s pharmacologic properties as a partial opioid agonist, along with its ceiling effect, confer a significantly decreased overdose and respiratory depression risk compared to full agonists [54-56]. As a result, diverted buprenorphine may displace higher-risk opioid exposures and improve outcomes at the population level [54-56]. While diversion should be averted, its consequences are unique with buprenorphine and should not dissuade family physicians from MOUD engagement [54-56].

Opioid use disorder is a chronic and relapsing condition. As such, opioid use and abnormal urine drug assays, especially early in the management course, are anticipated and should not signal treatment failure or the need to exclude patients from further interventions [57-58]. Dismissal following early lapses has been associated with poorer outcomes compared to continued patient engagement with MOUD [57-58]. Therefore, transient relapse should prompt consideration of dose optimization, reassessment of support needs, reinforced harm-reduction strategies, and other therapeutic adjustments rather than termination from the treatment program [57-58].

In addition to structured monitoring and relapse-tolerant care models, pharmacologic overdose mitigation remains an integral component of longitudinal opioid use disorder management. Several solo naloxone preparations, such as intranasally administered naloxone, are available for the acute management of opioid overdose [2-4]. These formulations should also be prescribed for individuals with opioid use disorder, including patients receiving buprenorphine, a history of overdose, chronic opioid pain management, sleep-disordered breathing, concurrent use of opiates and benzodiazepines, and anyone at increased risk for opioid overdose to mitigate this risk [2-4]. Previously, the CDC stipulated that as-needed naloxone for overdose treatment should be provided to patients with chronic pain who are on at least 50 morphine milligram equivalents (MME) or more of daily opioid doses; however, new guidance does not set a strict dose threshold and is inclusive of all patients at risk for opioid overdose, including patients on lower daily doses [2-4,33].

Primary care versus specialty care and other care settings

Practitioners, such as addiction and general psychiatrists, have traditionally maintained a monopoly on the treatment of opioid-use disorder. The expansion of the field into primary care has prompted questions about the appropriateness of family physicians and other non-specialist providers independently undertaking the challenge of opioid dependence management. One 2020 cross-sectional investigation of Carolina Medicaid claims demonstrated that primary care providers provided comparable quality of care to specialists for opioid use disorder and were even more likely to follow guidelines for certain measures [59]. Korthuis and colleagues conducted a 2017 randomized controlled trial investigating multiple office-based models for pharmacologic management of opioid use disorder and found that family medicine and other primary care-based treatment systems provided parallel quality to specialty-driven interventions [60]. Several investigations into patient perspectives on primary care-provided MOUD report higher satisfaction compared to traditional specialist-directed therapy, citing decreased stigma, improved convenience and accessibility, continuity of care, and stronger therapeutic relationships [61-62].

While the literature supports the idea that family medicine practitioners can provide safe and effective MOUD, questions concerning the necessity of psychiatric consultation are also posed. A 2011 Cochrane review by Amato et al. found that adding psychosocial support to standard pharmacologic MOUD interventions did not add any additional benefit [63]. A 2006 randomized controlled trial by Fiellin et al. compared pharmacologic-focused primary care opioid use disorder management with varying levels of additional supportive counseling interventions and found no difference in outcomes between the groups [64]. While specialty consultation and behavioral interventions may be prudent, especially in complex or complicated clinical scenarios, they are not a requirement for the initiation and maintenance of buprenorphine therapy for opioid use disorder among family medicine providers [63-65]. Importantly, these findings answer one of the more commonly reported barriers for family physicians prescribing MOUD. In our survey, 50% of respondents stated that the lack of availability of community specialists in substance use disorder was a barrier to initiating MOUD in their practice, and 37.5% of those surveyed noted that insufficient mental health support services in their area were a limiting factor. The current literature and MOUD guidelines should provide assurance and comfort to family physicians that consultants and specialty services are not a necessity for undertaking pharmacologic opioid use disorder treatment in their practices and that outcomes are not affected by this perceived barrier.

While this review focuses on clinic-oriented family medicine-based pharmacologic interventions for opioid use disorder, research has similarly been conducted on emergency department and inpatient implementation of MOUD. D’Onofrio et al. demonstrated that emergency department-initiated MOUD with referral to outpatient care is effective and associated with improved treatment retention and reduced illicit opioid use [66]. Fockele et al. and Duber et al. have studied multiple models for emergency department buprenorphine initiation with an evaluation of common barriers and an emphasis on developing optimized transitions of care to long-term chronic outpatient management programs [67-68]. Incze et al. evaluated the importance of engaging patients with opioid use disorder while hospitalized, the opportunity to begin MOUD, and methods to successfully facilitate transitions to longitudinal care after discharge [69]. These investigations have confirmed the value of expanding MOUD not just among primary care physicians but throughout varied clinical settings where patients with opioid use disorder are encountered [66-69]. State-level epidemiologic data from Louisiana further support the importance of emergency departments as critical settings for opioid use disorder interventions, as opioid-related emergency department visits have increased substantially in parallel with opioid-related mortality over the last decade [23]. This pattern, along with non-increasing hospital admissions and low primary care MOUD utilization, corroborates the assertion that emergency departments serve an expanding role as primary points of contact for opioid-related crises and MOUD induction opportunities, but difficult transitions to inpatient and outpatient continuity care, including lack of referral options, may limit successful outcomes [66-69].

Education as a foundational barrier

Insufficient education or background in MOUD was reported as a barrier for 48% of survey takers. Education is not only its own isolated limitation, but it also underpins several other perceived barriers associated with misconceptions and outdated information on MOUD. While it is true that substance use disorder has traditionally represented a gap in primary care training, contemporary family medical program requirements include instruction in opioid use disorder and pharmacologic management of opioid dependence as mandated by the ACGME [70-74]. Congruently, newer family medicine providers and recent residency graduates are more likely to prescribe buprenorphine in clinical practice than mid- and late-career family physicians, reflecting the importance of education on real-world adoption of MOUD implementation [70-74]. Comprehensive evidence-based education may serve as a unifying solution to multiple reported impediments to buprenorphine prescribing and should be a focus of barrier mitigation among family physicians. Numerous high-quality post-residency educational resources are readily accessible to practicing family physicians, including structured curricula from the ASAM, mentorship-based training through the Providers Clinical Support System (PCSS), and federal guidance such as the Substance Abuse and Mental Health Services Administration (SAMHSA) Treatment Improvement Protocol 63 [75-77]. Primary care-focused addiction education can be accessed through the AAFP MOUD education modules [78]. A list of several available established educational resources is summarized (Table 3) [23,75-79].

**Table 3:** Educational resources for medication for opioid use disorder (MOUD) Table created by the author from compiled educational resources [23,75-79].

Resource	Sponsoring organization	Educational focus	Format	Accessibility
American Society of Addiction Medicine (ASAM) Core Curriculum in Addiction Medicine [75]	ASAM	Comprehensive addiction medicine education, including MOUD	Online modules, continuing medical education (CME)	Paid; CME available
Providers Clinical Support System [76]	SAMHSA-funded initiative	Buprenorphine prescribing, case-based learning, clinical mentorship	Webinars, mentoring, CME	Free
Substance Abuse and Mental Health Services Administration (SAMHSA) Tip 63: Medications for Opioid Use Disorder [77]	SAMHSA	Evidence-based guidance on MOUD selection, initiation, and management	Practice guideline/educational reference	Free
American Academy of Family Physicians (AAFP) Medication for Opioid Use Disorder (MOUD) Educational Modules [78]	AAFP	Primary care-focused MOUD education	CME modules, clinical resources	Free with member access
Centers for Disease Control and Prevention (CDC) Opioid Prescribing and Opioid Use Disorder Training [79]	CDC	Opioid risk mitigation, OUD recognition, linkage to treatment	Online CME	Free
State-Supported MOUD Training Programs [23]	State health departments/Medicaid	Local prescribing guidance, regulatory navigation, practice support	Workshops, online modules	Free or low cost

Informed by the possibility of addressing multiple modifiable barriers through targeted dissemination of MOUD education, accessible, practice-oriented educational opportunities should be developed and made available to Louisiana primary care physicians.

Financial and reimbursement considerations

Financial and reimbursement concerns are often cited as barriers to MOUD clinical integration, and 12.5% of family physicians in our cohort reported these as potential obstacles to buprenorphine prescribing. Perceptions of inadequate payment, administrative complexity, and payer-imposed requirements such as prior authorizations are drivers of physician hesitancy and represent real deterrents to office-based primary care MOUD [80-82]. Still, emerging evidence suggests that these barriers are mostly structural and not intrinsic to buprenorphine prescribing itself [80-82]. The clinical, administrative, and time burdens of buprenorphine prescribing have been largely alleviated by several policy changes, including the removal of the X-waiver, reductions in prior authorization requirements, and the development of home-based induction models [80-82]. Buprenorphine visits can now be reimbursed using standard evaluation and management billing codes and require no more time than typical chronic care management encounters [80-82]. Office-based MOUD visits also frequently meet high-complexity billing criteria due to the high-risk nature of opioid use disorder, the use of controlled substances, and the need for ongoing risk assessment, monitoring, and care coordination [80-82]. Under-recognition of this complexity may lead to under-billing and contribute to perceived reimbursement inadequacy [80-82]. Newer payment-focused analyses propose aligning reimbursement with visit complexity, expanding telehealth coverage, and adopting alternative payment models to improve sustainability [80-83]. Financial modeling has demonstrated that team-based care paradigms incorporating nursing or care-management support can make office-based buprenorphine treatment economically viable in primary care [80-83]. Taken together, these findings indicate that while reimbursement barriers are real, many of them are exaggerated or modifiable through targeted policy and workflow interventions.

Practice partner hesitation

While concerns over practice partner resistance to implementing MOUD were one of the least reported barriers and only noted by a subset of our respondents, 10%, it remains a real and consequential limitation for potential buprenorphine prescribers. In small and independently governed primary care practices, even passive partner resistance may serve a gatekeeping function, as the scope of service is often shaped by informal consensus rather than individual clinician preference [2-4]. Prior investigations into MOUD adoption barriers similarly identified a lack of institutional or colleague support as a key determinant of non-prescribing, independent of physician knowledge or training [8-16]. Partner’s hesitations likely reflect a culmination of multiple barriers, including workflow disruption and staff burden from time-consuming visits, medicolegal and regulatory concerns, and anticipated changes in clinic culture [8-16]. While education likely serves a fundamental function in addressing practice partners’ concerns, structured organizational strategies, including pilot implementation by a single provider, clearly defined protocols, transparent financial monitoring demonstrating stable, longitudinal high-yield encounter billing, and a framing of MOUD as routine chronic disease management, may help encourage acceptance of buprenorphine initiatives in group practices [8-16].

Concerns regarding clinic disruption and patient mix

The survey barrier most commonly affirmed in our questionnaire was concern over attracting drug users and disruptive patients to practices, and was reported by 54% of respondents. While boundary establishment through patient-physician contracts, including clear behavioral expectations and discharge policies for diversion or threats, is recommended, this answer does not fully address the real and complex problem of potential practice ecosystem destabilization [2-4,84]. A nuanced perspective on this issue asserts that individuals with opioid use disorder are already part of a practice whether identified, treated, or untreated. Untreated patients are more likely to present to a clinic via unscheduled, episodic, crisis-driven encounters, whereas treated patients can transition to more stabilized and structured longitudinal management, less prone to disruptions [84]. While research on practice disruption is rare, one qualitative analysis demonstrated that provision of opioid substitution therapy in general practice does not substantially disrupt waiting room ambience or clinic operations [84]. In this investigation, most patients were unaware of the opioid substitution prescribing practices of the offices, disruptions were rare in both opioid substitution and non-opioid substitution facilities, and only a small percentage of patients reported being likely to change practices if their provider participated in MOUD [84]. Still, this barrier is uniquely difficult as it involves emotional and anticipatory fears shared by prescribers and the clinic staff collective, and represents a stigmatized issue which individuals may be hesitant to discuss or engage with. Given its complexity, this barrier may necessitate future research examining clinic-level experiences following MOUD implementation to inform strategies to address fears and stigma without dismissing them.

Simplified reference for new MOUD prescribers

**Figure 6:**
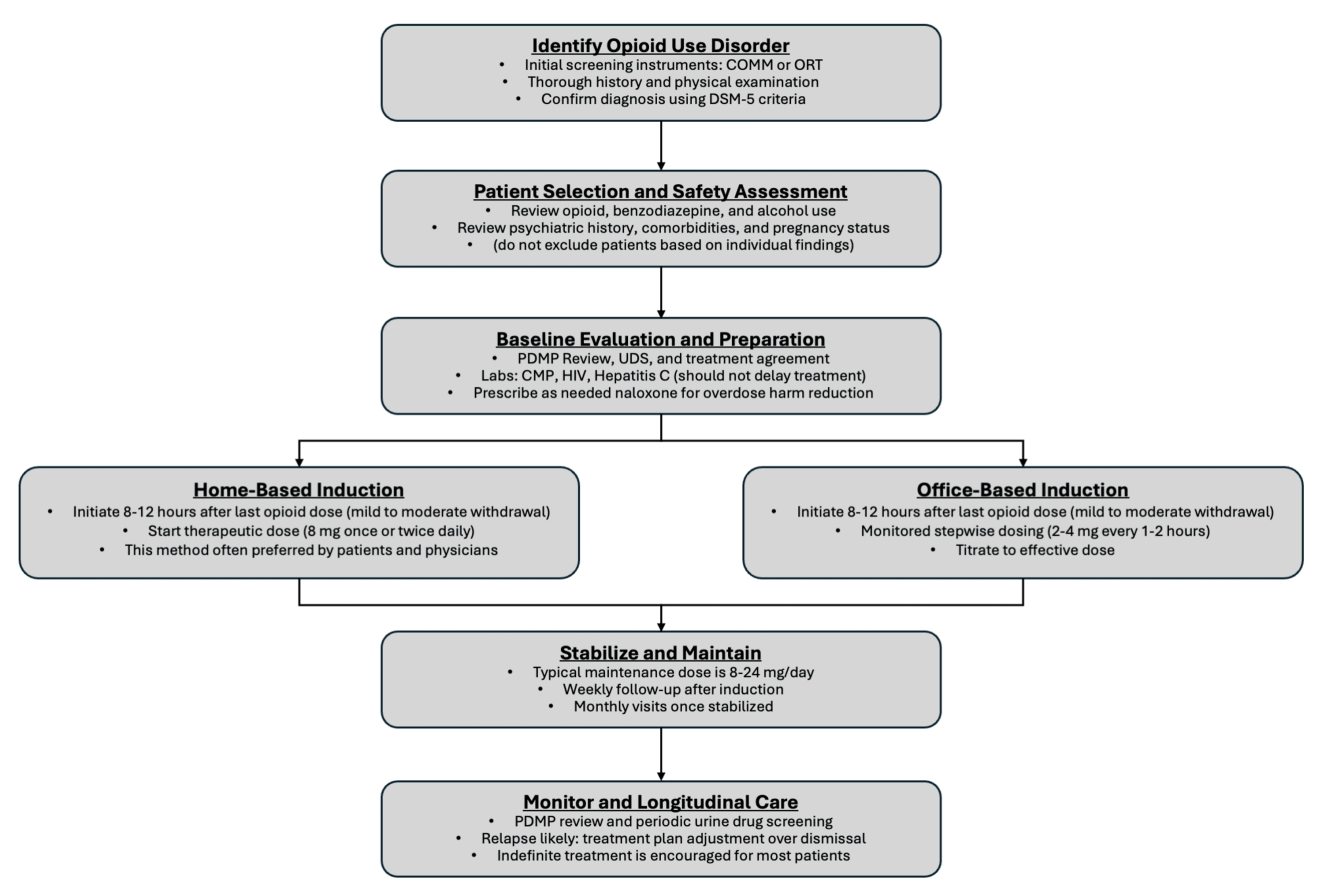
Primary care algorithm for buprenorphine treatment of opioid use disorder Simplified primary care reference algorithm for buprenorphine treatment of opioid use disorder, created by the author using PowerPoint (Microsoft® Corp., Redmond, WA) and Adobe Acrobat (Adobe Inc., San Jose, CA) and informed by established clinical guidance [2-4,17-22,33,37]. Diagnostic criteria and withdrawal assessment tools referenced are publicly available and cited in the main text [17-22].

In response to the complex and multifactorial challenge of integrating MOUD into practice, a reference algorithm is provided as a simplified distillation of the practical principles of buprenorphine prescribing. The algorithm is designed as a pragmatic reference to support family physicians who may be hesitant to initiate MOUD and is not intended to function as a comprehensive guideline or institutional protocol. Rather, it serves to illustrate an approachable pathway for integrating buprenorphine treatment into routine primary care practice while allowing for clinical judgment and local resource considerations (Figure 6).

Limitations

This study has several limitations that should be considered when interpreting its findings. First, as a pilot, cross-sectional survey, the results are exploratory and descriptive in nature and are not intended to establish causality or be broadly generalizable beyond the queried population. Second, participation was limited to LAFP members, which may not fully represent all family physicians practicing in Louisiana, including those who are not members of the organization or who practice in other clinical settings. Third, responses were self-reported and therefore susceptible to recall and social desirability bias, potentially influencing how barriers were perceived or reported. Fourth, the survey design allowed respondents to select multiple barriers, which limits direct comparison between individual factors and precludes formal statistical inference.

Additionally, in order to preserve respondent anonymity, demographic and practice-level data were not collected, restricting the opportunity to examine associations between perceived barriers and physician characteristics. Despite these limitations, this pilot study provides crucial preliminary insight into barriers to buprenorphine prescribing among Louisiana family physicians and potentially identifies areas for targeted educational, structural, and policy-level interventions.

## Conclusions

In this pilot survey of Louisiana family physicians, most respondents did not prescribe buprenorphine for opioid use disorder despite practicing in a state with a substantial burden of opioid-related morbidity and mortality. Reported barriers to prescribing were multifactorial and centered primarily on time constraints, educational gaps, perceived clinic disruption, and limited access to mental health and specialty support services, rather than concerns about medication need, safety, or effectiveness. These findings suggest that many barriers to buprenorphine prescribing in family medicine are potentially modifiable through targeted education, workflow support, and system-level interventions.

Family physicians are uniquely positioned to expand access to MOUD, particularly in communities where specialty addiction services are limited. Addressing the barriers identified in this study may facilitate broader integration of buprenorphine treatment into primary care and improve access to evidence-based interventions for patients with opioid use disorder. Future studies should build upon these exploratory findings with larger, longitudinal investigations to evaluate the impact of targeted interventions on buprenorphine prescribing rates and patient outcomes in primary care settings.

## Figures and Tables

**Figure 7:**
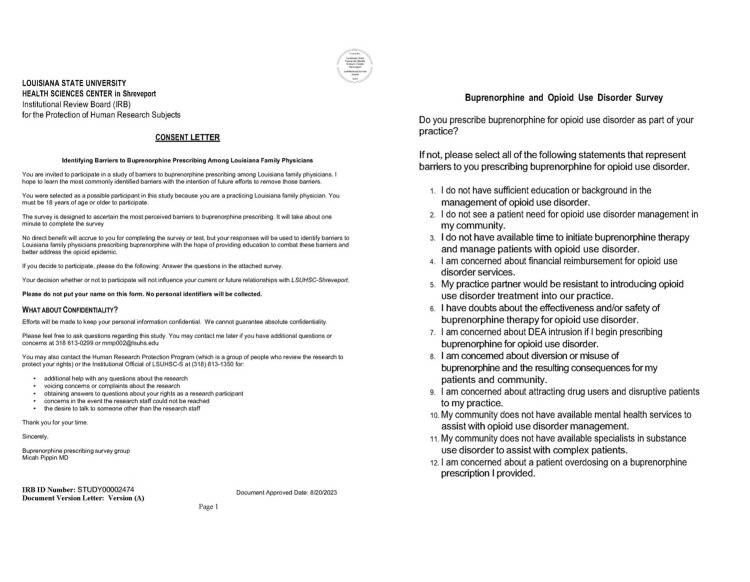
Buprenorphine prescribing barriers survey instrument Figure created by the author using Microsoft Word (Microsoft® Corp., Redmond, WA).
